# Node-RADS in cervical cancer: a multi-reader agreement and diagnostic performance study

**DOI:** 10.1186/s40644-026-01072-2

**Published:** 2026-06-17

**Authors:** Shifang Tan, Xueyan Liu, Bairu Li, Tingting Bao, Lingjie Zhang, Zhexuan Yang, Shaomin Li, Tian Ren, Meiying Cheng, Junjie Liao, Xiaoan Zhang, Xin Zhao

**Affiliations:** 1 Department of Radiology, The Third Affiliated Hospital of Zhengzhou University, Zhengzhou, 450052 People’s Republic of China; 2 Department of Information, The Third Affiliated Hospital of Zhengzhou University, Zhengzhou, 450052 People’s Republic of China; 3 Tianjian Laboratory of Advanced Biomedical Sciences, Institute of Advanced Biomedical Sciences, Zhengzhou University, Zhengzhou, 450000 People’s Republic of China

**Keywords:** Ervical cancer, Node-RADS, Agreement, MRI, Lymph node staging

## Abstract

**Objectives:**

To evaluate the agreement and diagnostic performance of the Node Reporting and Data System 1.0 (Node-RADS) for preoperative lymph node staging in cervical cancer across readers with different experience levels.

**Methods:**

This retrospective study enrolled 439 consecutive cervical cancer patients who underwent preoperative MRI and lymph node dissection. Target nodes were pre-specified by a most experienced consultant radiologist to unify the assessment objects. Four readers (two senior with 9–11 years of experience, two junior with 3–5 years of experience) independently assigned Node-RADS scores, blinded to histopathology. Inter-reader agreement and between-group agreement were assessed using weighted and Cohen’s kappa. Diagnostic performance was evaluated against histopathology as reference standard.

**Results:**

Senior readers achieved near-perfect agreement for Node-RADS scores (k = 0.988), nodal status (k = 0.959) and all individual morphological parameters (k = 0.847–0.967). Junior readers showed moderate to substantial agreement (k = 0.554–0.754). Although junior readers requiring consensus more frequently (7.1% vs. 1.4%, *p* < 0.01), the between-group agreement for nodal status was substantial (k = 0.783). For nodal metastasis detection, senior readers demonstrated higher diagnostic performance (AUC 0.868; sensitivity 76.4%; specificity 97.3%) compared to junior readers (AUC 0.758; sensitivity 54.6%; specificity 97.0%), and both groups presented favorable diagnostic efficacy overall.

**Conclusions:**

Inter-reader agreement for nodal status was almost perfect among senior readers and substantial among junior readers. Diagnostic performance was slightly better in the senior group, suggesting an influence of reader experience.

**Supplementary Information:**

The online version contains supplementary material available at 10.1186/s40644-026-01072-2.

## Introduction

Cervical cancer remains a significant global public health challenge. According to the GLOBOCAN 2022 database, there were approximately 660,000 new cases and 350,000 deaths occurred worldwide in 2022, making it a leading cause of both cancer incidence and mortality among women in numerous countries [1, 2].

Accurate staging, guided by the International Federation of Gynecology and Obstetrics (FIGO) system, is fundamental to determining optimal treatment strategies, ranging from surgery and chemoradiation for early-stage disease to systemic or palliative approaches for advanced cases. In this context, the presence and extent of lymph node metastasis (LNM) constitute one of the most critical prognostic factors, directly impacting patient survival and guiding clinical decisions on whether to intensify or reduce treatment regimens [3–5].

Although multiple imaging modalities are available for evaluating LNM, radiologic assessment of the lymphatic system remains challenging due to its complex anatomy and physiology, with each technique possessing inherent potentials and limitations [6]. Consequently, while pelvic MRI tailored for cervical evaluation is considered the best imaging approach for assessing local extent, it is widely recognized that conventional MRI diagnosis of LNM in cervical cancer suffers from low sensitivity, which can lead to under-staging and potentially inappropriate treatment planning [7, 8].

**Fig. 1:**
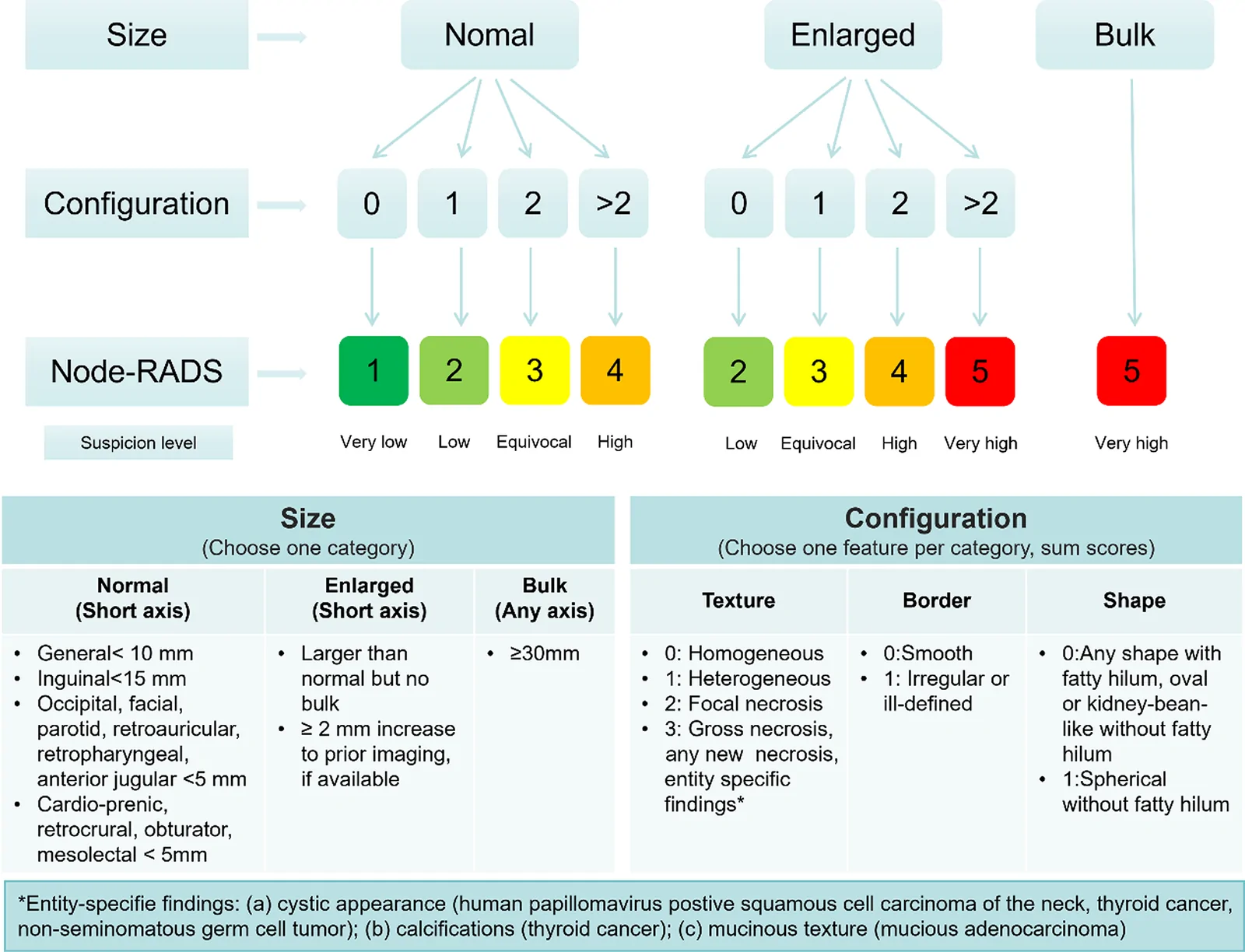
Node-RADS scoring flowchart. (modified from the original article [9])

Introduced in 2021 by Elsholtz FHJ et al., the Node Reporting and Data System 1.0 (Node-RADS 1.0) is a standardized scoring system developed to address the lack of consensus and standardization in radiological lymph node assessment across various cancer types [9]. This system synthesizes size and configuration criteria into a five-point scoring system (Fig. 1), with ascending scores indicating a higher probability of malignancy. In the context of lymph node staging, Node-RADS 1 and 2 should be reported as N (−) and Node-RADS 4 and 5 as N (+). The decision how to report Node-RADS 3 should depend on the characteristics of the primary tumor [9].

To date, research on Node-RADS has primarily focused on evaluating its diagnostic performance and inter-observer agreement. Studies in cancers such as breast, rectal, and bladder carcinoma have reported variable results regarding its diagnostic accuracy and inter-reader agreement [10–15]. In contrast, data on its application in cervical cancer are notably limited, necessitating further exploration. Therefore, the present study aims to evaluate the inter-reader agreement and diagnostic accuracy of Node-RADS for the preoperative nodal staging of cervical cancer using MR images.

## Methods

### Patients’ population

The institutional review board of the Third Affiliated Hospital of Zhengzhou University approved this retrospective study (2023-252-01) and granted a waiver of informed consent due to the utilization of anonymized data. Our study conformed to the Declaration of Helsinki on Human Research Ethics standards.

**Fig. 2:**
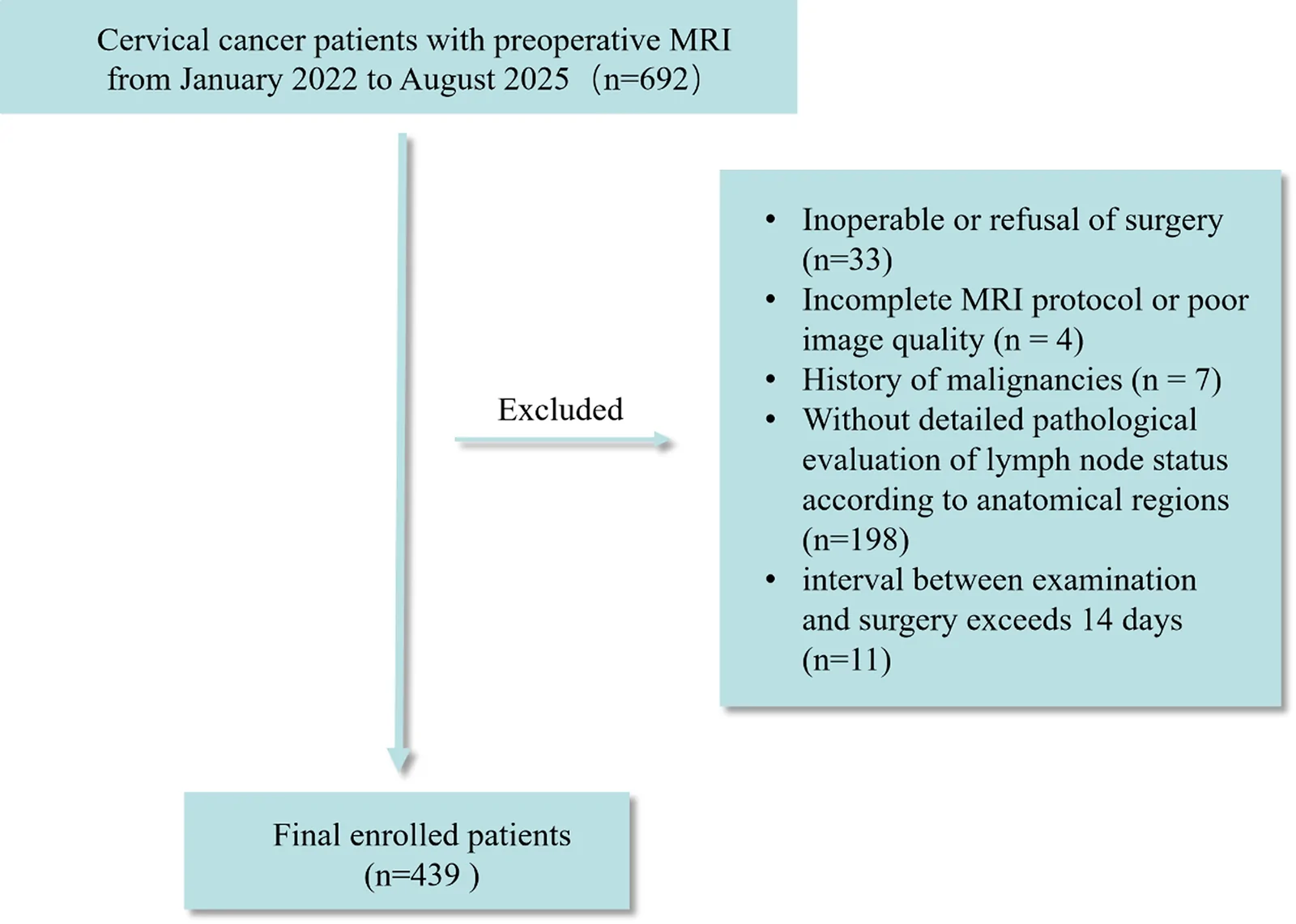
The flowchart of patient selection

We consecutively enrolled patients with histopathologically confirmed cervical cancer at our institution between January 2022 and August 2025. Inclusion Criteria: (a) Patients who underwent contrast-enhanced pelvic MRI for preoperative staging; (b) Patients who underwent radical hysterectomy with systematic pelvic lymphadenectomy, with or without para-aortic lymphadenectomy; (c) An interval of less than 14 days between the MRI examination and the radical surgery; (d) Availability of pathological assessment of pelvic lymph nodes (PLNs) based on anatomical subregions. Exclusion Criteria: (a) Patients with multiple primary tumors or a prior malignancy; (b) Incomplete MRI series or non-diagnostic image quality; (c) Incomplete histopathological report. A detailed flow diagram of patient selection is presented in Fig. 2.

### MRI acquisition protocol

MRI examinations were conducted using 3T scanners (GE Healthcare SIGNA Pioneer and Siemens Healthcare Skyra) equipped with an 18-channel abdominal phased array coil. The imaging protocol included: Axial and sagittal T2-weighted imaging (T2WI) without fat saturation, axial and coronal fat-saturated T2WI (FS-T2WI), axial in- and opposed-phase T1-weighted imaging (T1WI), axial diffusion-weighted imaging (DWI), and three-dimensional dynamic contrast enhanced MRI (DCE-MRI), acquired as a FS-T1WI sequence before and after intravenous contrast administration. Detailed acquisition parameters are provided in the supplementary materials (Table S1).

### Node-RADS assessment

Node-RADS assessments were conducted on the Picture Archiving and Communication System (PACS). To ensure that all readers evaluated the same lymph nodes, target lymph nodes were pre-specified prior to the reader study. First, an experienced consultant radiologist (R0) specializing in gynecologic imaging with 19 years of experience, who did not participate in the subsequent inter-reader agreement assessment, independently reviewed the preoperative MRI scans of all enrolled patients. For each patient, R0 annotated up to two target lymph nodes according to the following criteria: the primary target was the lymph node with the largest short-axis diameter within the pelvis; a secondary target (if present) was a lymph node considered more suspicious than the largest one based on morphological abnormalities (e.g., necrosis, irregular or ill-defined margins). The anatomical location of each target lymph node was documented on the case report form, this documentation also facilitated subsequent region-by-region correlation with pathological findings.

Subsequently, four radiologists with varying levels of experience were selected as readers: two senior readers (R1 and R2, with 11 and 9 years of pelvic imaging experience, respectively) and two junior readers (R3 and R4, with 5 and 3 years of experience in the same domain). All four readers independently and collectively reviewed the original Node-RADS publication by Elsholtz et al. [9], after which the most experienced radiologist (R0) led a structured training session comprising lectures and case-based discussions.

**Fig. 3:**
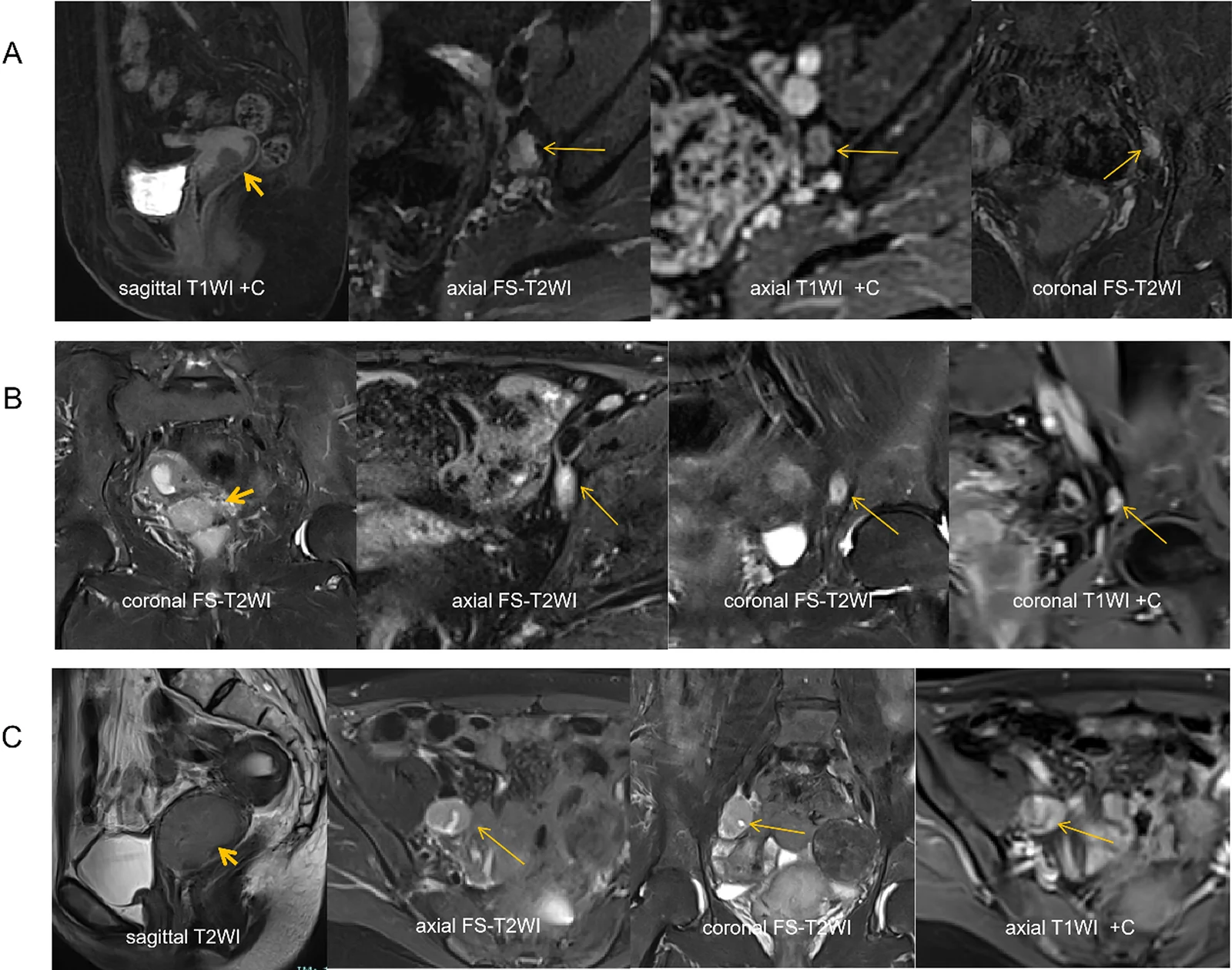
Representative examples of Node-RADS scoring. (**A**) A 56-year-old patient with squamous cell carcinoma of the cervix. The short arrow indicates the mildly enhancing cervical mass. The long arrow indicates the target lymph node (short-axis diameter 0.7 cm), which shows heterogeneous texture, irregular margins, and a kidney-bean shape. The node was assigned a Node-RADS score of 3. (**B**) A 79-year-old patient with squamous cell carcinoma of the cervix. The short arrow indicates the cervical mass. The target lymph node (long arrow, short-axis diameter 0.5 cm) exhibits heterogeneous texture, smooth margins, and a kidney-bean shape, corresponding to a Node-RADS score of 2. (**C**) A 53-year-old patient with squamous cell carcinoma of the cervix. The short arrow indicates the cervical mass. The target lymph node (long arrow, maximum diameter 3.1 cm) shows focal necrosis, a spherical shape without a fatty hilum, and was assigned a Node-RADS score of 5

Node-RADS scoring was performed according to the established recommendations (as shown in Fig. 1), with representative imaging examples provided in Fig. 3. For patients with multiple target PLNs, the highest Node-RADS score among them was assigned as the final nodal score for that patient and was used for the subsequent analyses. While all readers were informed of the presence of cervical cancer, they remained blinded to the final histologic type, tumor grade, and any additional clinical or pathological details.

### Lymph node evaluation

Based on the assigned scores, each examination was classified as N- or N + as follows: Node-RADS 1–2 were considered N-, and Node-RADS 4–5 were considered N+. For cases with a Node-RADS score of 3, classification was guided by MRI findings (patients with tumors ≤ 2 cm and no parametrial or vaginal invasion were classified as N–, while those with tumors > 2 cm or evidence of parametrial or vaginal invasion were classified as N+). Discrepancies in nodal staging between the two readers of each subgroup (R1 vs. R2, R3 vs. R4) were solved by consensus.

All enrolled patients underwent radical hysterectomy with systematic pelvic lymphadenectomy, with or without para-aortic lymphadenectomy. PLNs were surgically harvested from the bilateral obturator, external iliac, internal iliac, and common iliac regions, and were submitted separately according to these anatomical subregions for histopathological examination. The histopathological reports served as the reference standard. For each patient, the total number of lymph nodes retrieved and the number of metastatic nodes were recorded. Micrometastases (0.2–2 mm) and Macrometastases (> 2 mm) were documented where applicable. Additional histopathological characteristics (including tumor histology type, lymphovascular space invasion, parametrial involvement, vaginal invasion) were also extracted from the pathology reports.

### Statistical analysis

Statistical analyses were performed using SPSS Statistics Software (version 27.0, IBM, Armonk, NY, USA). Continuous variables were expressed as mean ± standard deviation or median with interquartile range based on their distribution. Categorical variables were presented as frequencies and percentages.

Inter-reader agreement was assessed within each experience subgroup for lymph node size and morphological features (texture, margin, shape) across all target nodes, as well as for overall Node-RADS scores and final nodal status (N-/N+) assigned at the patient level. Between-group agreement was evaluated for final nodal status based on the consensus readings of the two experience groups. The weighted kappa coefficient (with quadratic weights) was used for ordered categorical variables, and the Cohen’s kappa coefficient (simple kappa) was employed for dichotomous variables. All kappa values were reported with 95% confidence intervals (CIs).

With surgical pathology as the reference standard, the diagnostic accuracy of Node-RADS for LNM was evaluated for each experience group based on consensus status. Sensitivity, specificity, accuracy, positive predictive values (PPV), and negative predictive value (NPV) were calculated with 95% CIs. Receiver operating characteristic (ROC) curve analysis was conducted to estimate the area under the curve (AUC) for each group. Statistical significance was set at *p* < 0.05.

## Results

### Patient characteristics and pathological findings

A total of 439 patients were included, contributing 445 target PLNs for Node-RADS evaluation. The median age was 54 years (IQR, 45–60). Histopathological examination confirmed LNM in 110 of the 439 patients (25.1%). Among these, 65 pathological reports specified the size of metastatic lesions, revealing macrometastases in 60 cases (92.3%) and micrometastases in 5 cases (7.7%). The clinical and pathological characteristics of the study cohort are summarized in Table 1.

**Table 1:** Clinical and pathological characteristics of the study population

Characteristics	Total (*n* = 439)	*N*+ (*n* = 110)	*N*- (*n* = 329)
Age (year)	54 (45,60) *	53 (44,59) *	54 (45,60) *
Tumor MD (cm)	3.1 (2.0,4.3) *	4.2 (3.2,5.5) *	2.7 (1.6,3.8) *
Tumor stage (FIGO 2018)			
Ⅰ	254 (57.9%)	0 (0.0%)	254 (77.2%)
Ⅱ	72 (16.4%)	0 (0.0%)	72 (21.9%)
Ⅲ	104 (23.7%)	102 (92.7%)	2 (0.6%)
Ⅳ	9 (2.1%)	8 (7.3%)	1 (0.3%)
Lymphovascular space invasion			
Negative	202 (46.0%)	14 (12.7%)	188 (57.1%)
Positive	237 (54.0%)	96 (87.3%)	141 (42.9%)
Parametrial involvement			
Negative	388 (88.4%)	74 (67.3%)	314 (95.4%)
Positive	51 (11.6%)	36 (32.7%)	15 (4.6%)
Vaginal invasion (%)			
Negative	327 (74.5%)	63 (57.3%)	264 (80.2%)
Positive	112 (25.5%)	47 (42.7%)	65 (19.8%)
Tumor histology type			
Squamous cell carcinoma	328 (74.7%)	77 (70.0%)	251 (76.3%)
Adenocarcinoma	89 (20.3%)	31 (28.2%)	58 (17.6%)
Adenosquamous carcinoma	16 (3.6%)	0 (0.0%)	16 (4.9%)
Other rare types	6 (1.4%)	2 (1.8%)	4 (1.2%)

*N+* target lymph node metastasis positive, *N-* target lymph node metastasis negative, *FIGO* International Federation of Gynecology and Obstetrics, * Data are medians, with IQRs in parentheses

### Inter-reader agreement within each group

In the senior reader group (R1 and R2), inter-reader agreement for PLN size and morphological features was almost perfect. Specifically, the (weighted/Cohen’s) kappa values for size, texture, margins, and shape were 0.962 (95% CI: 0.934–0.989), 0.967 (95% CI: 0.950–0.983), 0.847(95% CI: 0.759–0.936); and 0.884 (95% CI: 0.820–0.949), respectively. Concordance rates for these parameters were 98.4%, 95.3%, 97.5%, and 97.3%, respectively. For Node-RADS score, weighted kappa was 0.988 (95% CI: 0.982–0.993), when dichotomized into nodal status (N- vs. N+), Cohen’s kappa was 0.959 (95% CI: 0.926–0.992), both reflecting almost perfect consistency.

In the junior reader group (R3 and R4), inter-reader agreement for PLN size and morphological features ranged from moderate to substantial. Specifically, the (weighted/Cohen’s) kappa values for size, texture, margins, and shape were 0.754 (95% CI: 0.675–0.834), 0.554 (95% CI: 0.466–0.642), 0.563 (95% CI: 0.412–0.714), and 0.663(95% CI: 0.542–0.784), respectively. Concordance rates for these parameters were 92.4%, 62.0%, 94.4%, and 94.4%, respectively. For Node-RADS score, weighted kappa coefficient was 0.737 (95% CI: 0.673-0.800), when dichotomized into nodal status (N- vs. N+), Cohen’s kappa coefficient was 0.729 (95% CI: 0.638–0.819), both indicating substantial agreement. The results of the agreement analysis for Node-RADS scoring and nodal status are presented in Table 2.

**Table 2:** Results of the inter-reader agreement for Node-RADS scoring and the nodal status

Group	Node-RADS score		Nodal status	
	weighted k (95% CI)	gradation	Cohen’s k (95% CI)	Gradation
Senior readers	0.988 (0.982–0.993)	almost perfect	0.959 (0.920–0.992)	almost perfect
Junior readers	0.737 (0.673-0.800)	substantial	0.729 (0.638–0.819)	Substantial

### Consensus requirements and between-group agreement

**Fig. 4:**
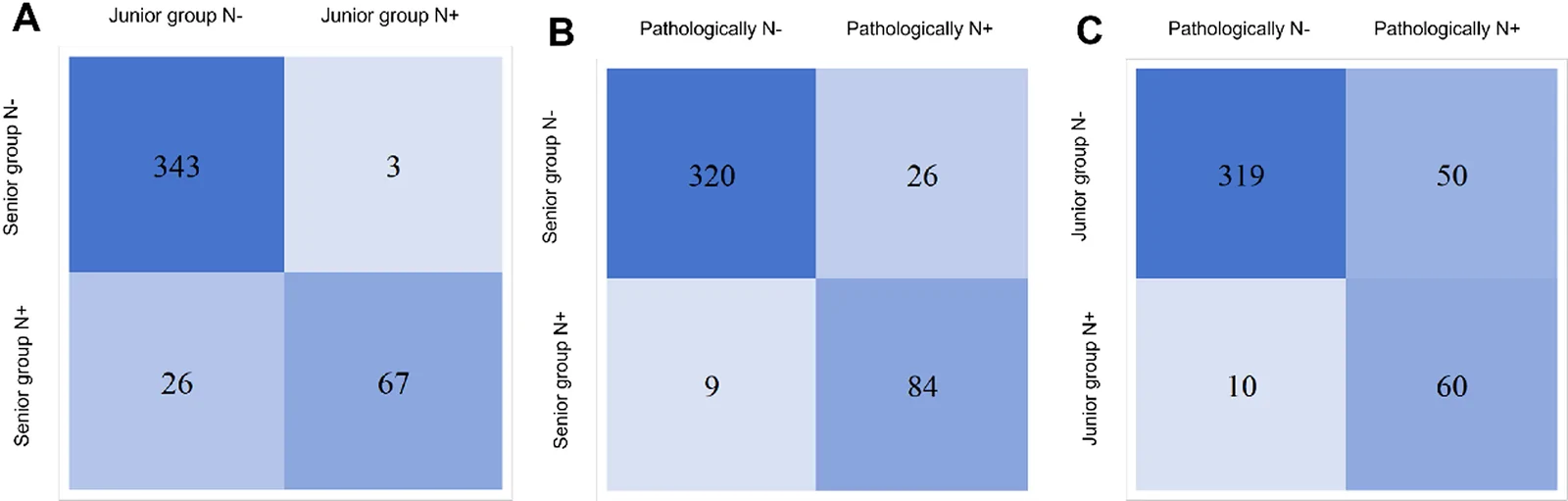
Heatmap of agreement in lymph node status (N–/N+) classification: (**A**) Senior reader group vs. junior reader group; (**B**) Senior reader group vs. pathological gold standard, (**C**) Junior reader group vs. pathological gold standard

Consensus due to initial disagreement between readers was required in 6 of 439 patients (1.4%) in the senior group and in 31 patients (7.1%) in the junior group (χ² = 17.64, *p* < 0.01). After consensus, the senior group classified 93 patients (21.2%) as N + and 346 (78.8%) as N-; the junior group classified 70 patients (15.9%) as N + and 369 (84.1%) as N-. Agreement between the consensus readings of the two groups for nodal status was substantial, with a Cohen’s kappa coefficient of 0.783 (95% CI: 0.707–0.858), and was visualized using a heatmap (Fig. 4A).

### Diagnostic performance against histopathology

The diagnostic performance of the two groups for lymph node status was evaluated against the histopathology gold standard, with the distribution of consistent/inconsistent cases visualized by heatmaps (Fig. 4B and C). For the senior reader group, the diagnostic indices were as follows: a sensitivity of 76.4%, specificity of 97.3%, accuracy of 92.0%, PPV of 90.3%, and NPV of 92.5%. The junior readers achieved a sensitivity of 54.6%, specificity of 97.0%, accuracy of 86.3%, PPV of 85.7%, and NPV of 86.5%. As shown in Table 3.

**Table 3:** Diagnostic performance of senior and junior groups for lymphnode status

Group	AUC (95%CI)	Sensitivity (95%CI)	Specificity (95%CI)	Accuracy (95%CI)	PPV (95%CI)	NPV (95%CI)
Senior readers	0.868 (0.819–0.917)	76.4% (0.676–0.833)	97.3% (0.949–0.986)	92.0% (0.891–0.942)	90.3% (0.826–0.948)	92.5% (0.892–0.948)
Junior readers	0.758 (0.697–0.818)	54.6% (0.452–0.635)	97.0% (0.945–0.983)	86.3% (0.828–0.892)	85.7% (0.757–0.921)	86.5% (0.826–0.896)

**Fig. 5:**
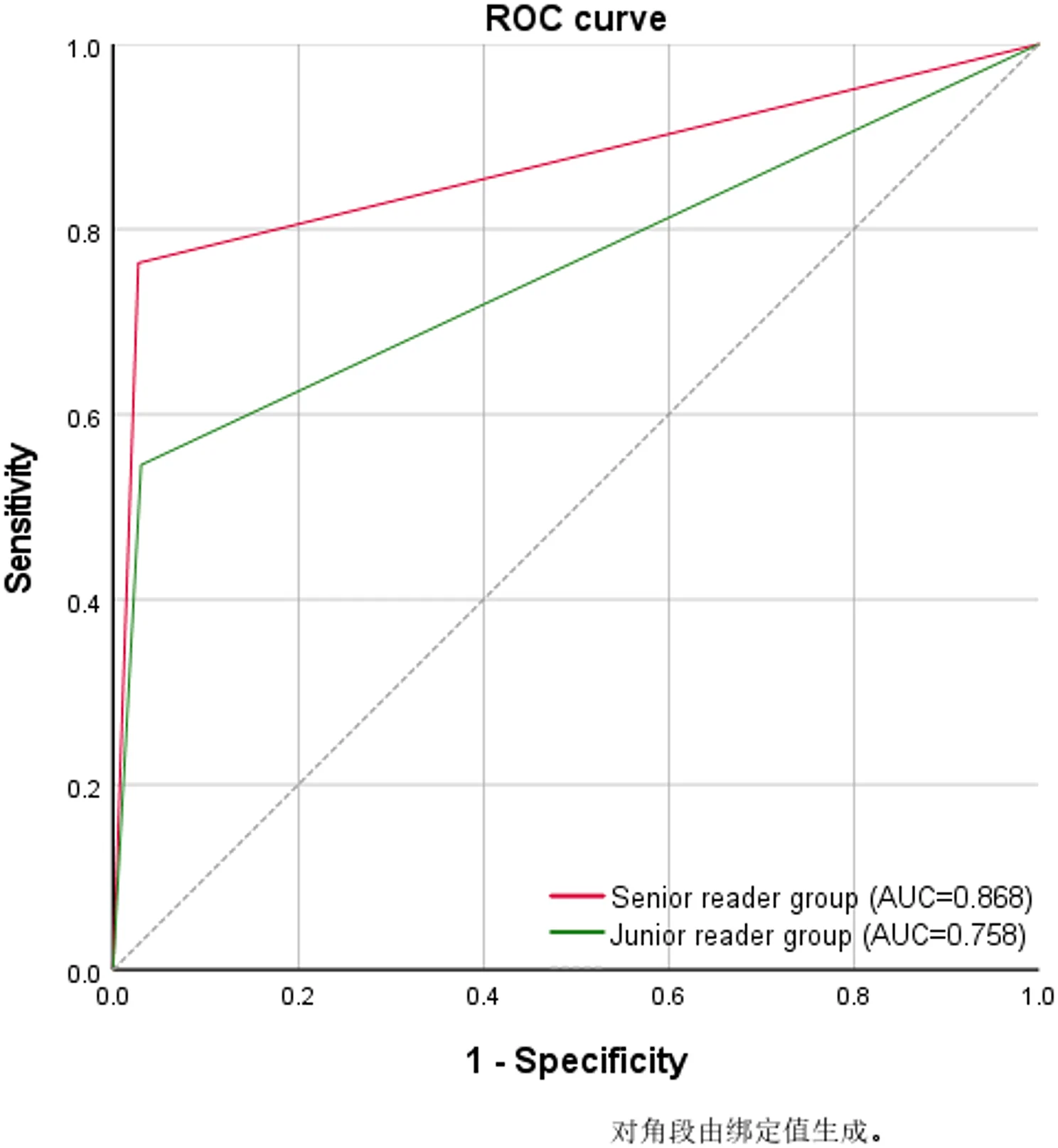
Receiver operating characteristic (ROC) curves of two groups’ discriminatory for lymph node status prediction

ROC curve analysis further quantified the two groups’ discriminatory ability for PLN status prediction (Fig. 5). The senior reader group had a higher AUC (0.868, 95% CI: 0.819–0.917) than the junior reader group (0.758, 95% CI: 0.697–0.818). This demonstrated that both groups had good diagnostic value for lymph node status assessment, with the senior readers showing superior discriminatory ability.

## Discussion

Since the introduction of Node-RADS, a growing number of studies have investigated its diagnostic performance across various types of tumors [10–15]. However, its application remains largely in the validation phase, and evidence specific to cervical cancer is limited. Currently, the available studies we have identified on Node-RADS for cervical cancer patients are limited to just two [16, 17], both with relatively small sample sizes at the patient level (81 and 68 patients, respectively), and focusing primarily on diagnostic performance. Recent systematic reviews have highlighted that existing Node-RADS studies are characterized by relatively small sample sizes, with particularly insufficient data on inter-observer reliability, underscoring the need for more studies comparing consistency across multiple readers with different levels of experience [18, 19]. We therefore conducted a large-scale retrospective study focusing on the agreement of Node-RADS for preoperative nodal staging in cervical cancer across readers with different levels of experience, along with a basic assessment of diagnostic performance. Our results showed that among experienced readers, Node-RADS achieved near-perfect inter-reader agreement and excellent diagnostic accuracy. Notably, even junior readers with limited experience in pelvic imaging demonstrated moderate-to-substantial agreement and acceptable diagnostic performance, though lower than those of their senior counterparts. These findings suggest that Node-RADS is a reasonably robust tool for nodal assessment in cervical cancer with potential for clinical applicability across different experience levels.

In this study, we used pre-specified target lymph nodes rather than allowing readers to select their own. This choice was made to separate two sources of variability that are often mixed in reader studies: finding the node and scoring it. When readers choose their own targets, it is difficult to tell whether disagreement comes from selecting different nodes or from scoring the same node differently. By pre-specified the nodes, our agreement results reflect the consistency of Node-RADS scoring itself, rather than differences in search patterns. This design also made it easier to match imaging findings with pathology by anatomical region. That said, the pre-specified process relies on a single expert’s judgment, which introduces some subjectivity. Future studies could use two experts for pre-annotation or include an independent detection phase to address this limitation. From this foundation, we then assessed inter-observer agreement across experience levels.

To obtain a comprehensive picture of reproducibility, we assessed inter-reader agreement not only for the Node-RADS score and nodal status, but also for each individual imaging parameter of lymph nodes (including size, texture, margins, shape) using weighted kappa or Cohen’s kappa, which provides a more rigorous evaluation. Our results revealed that senior readers achieved almost perfect agreement for both size and all morphological parameters (k range 0.847–0.967). In contrast, junior readers demonstrated moderate to substantial agreement across these parameters (k range 0.554–0.754), consistently lower than their senior counterparts, indicating that diagnostic experience influences the consistent interpretation of subtle imaging characteristics. Consistent with prior Node-RADS studies in renal [20] and gastric [14] cancer, we found that size yielded high and consistent inter-reader agreement in both groups, while morphological features, particularly texture and margins, showed relatively lower agreement among the junior group. This consistent observation across different tumor types further confirms that lymph node size is the most robust parameter in Node-RADS-based assessment, owing to its objective and quantifiable nature in contrast to the more subjective evaluation of morphological features.

For the final Node-RADS score and the nodal status, senior readers achieved near-perfect agreement (weighted k = 0.988, Cohen’s k = 0.959), while junior readers likewise attained substantial agreement (weighted k = 0.737, Cohen’s k = 0.729), suggesting that even less experienced readers can apply the Node-RADS system with reasonable consistency after targeted training. Moreover, although the junior group required consensus in a significantly higher proportion of cases, the two groups reached highly concordant final nodal status decisions after consensus (Cohen’s k = 0.783). The agreement levels in our study fall within the range reported in previous Node-RADS studies (k = 0.620–0.888) [10, 16, 21, 22]; however, those investigations focused primarily on diagnostic performance without stratifying readers or systematically assessing inter-group agreement. Compared with a recent study examining Node-RADS reproducibility across experience levels in endometrial cancer [23], our observed agreement was somewhat higher. This difference may be attributable to our use of pre-specified target lymph nodes, which eliminated variability in node selection and allowed a purer assessment of scoring consistency. The overall high consistency demonstrated in our study underscores the standardizing role of the Node-RADS system and supports its potential clinical utility in routine practice.

Most previous Node-RADS studies have used a fixed cutoff of ≥ 3 or ≥ 4 for nodal positivity. While practical, this does not fully reflect the original intent of the scoring system. As Elsholtz et al. [9] emphasized, interpretation of the equivocal Node-RADS 3 category should be guided by primary tumor characteristics. Following this principle, and supported by evidence that tumor size < 2 cm, no parametrial invasion, and FIGO stage < IB2 indicate low nodal metastasis risk in cervical cancer [24–26], we incorporated these clinical and imaging findings when assigning nodal status for Node-RADS 3 patients. This approach integrates the Node-RADS system with tumor biology while maintaining a coherent analytical structure. We view this as a practical solution, though not a formally validated standard; future studies specifically addressing outcomes of Node-RADS 3 patients will help refine the optimal approach.

Regarding diagnostic performance, the senior group achieved higher sensitivity, specificity, accuracy, and AUC (0.868) compared with the junior group, consistent with the well-recognized advantage of clinical experience in imaging interpretation. Importantly, junior readers still achieved favorable diagnostic efficacy, with an AUC of 0.758 and accuracy of 86.3%. A longstanding limitation of conventional MRI in lymph node assessment has been its relatively low sensitivity for detecting metastasis, reportedly 51–57% in previous studies [8, 27]. In our cohort, junior readers using Node-RADS achieved a sensitivity of 54.6%, in line with previously reported values, whereas senior readers showed a marked improvement, with sensitivity reaching 76.4%. These findings suggest that while Node-RADS may not fully overcome the inherent challenges of detecting nodal metastases in less experienced hands, its structured approach enables experienced readers to substantially enhance diagnostic sensitivity beyond conventional MRI. This underscores the value of Node-RADS as a standardized reporting tool that, when combined with reader expertise, can improve preoperative nodal staging in cervical cancer.

The study had several limitations. First, its retrospective design may carry risks of selection bias and dataset imbalance, and the single-center design makes all readers receiving the same standardized training, which may have resulted in higher agreement than would be observed in a multi-institutional setting. Second, although we employed a pre-specified strategy to ensure all readers evaluated the same target nodes, this approach relies on a single radiologist for node selection, which may introduce some degree of subjectivity. Finally, exact node-by-node pathological correlation was not achievable, which would require a prospective study with integrated collaboration across radiology, gynecology, and pathology [28].

In conclusion, our study demonstrates that Node-RADS is a reliable tool with good potential for standardized assessment for preoperative nodal staging in cervical cancer. Clinical experience exerts a certain impact on the identification of subtle lymph node imaging features and diagnostic performance, so targeted training on morphological feature recognition is recommended for less experienced radiologists.

## Supplementary Information

Below is the link to the electronic supplementary material.

Supplementary Material 1

## Data Availability

All data included in this study are available from the corresponding author or first author on reasonable request.

## Abbreviations

AUC: Area under the curve
FIGO: International Federation of Gynecology and Obstetrics
LNM: Lymph node metastasis
MRI: Magnetic resonance imaging
Node-RADS: Node Reporting and Data System
NPV: Negative predictive value
PLNs: Pelvic lymph nodes
PPV: Positive predictive values
ROC: Receiver operating characteristic
