# Supplementary material for: Node-RADS in cervical cancer: a multi-reader agreement and diagnostic performance study
Source: Cancer Imaging. 2026 Jun 17;26:113. doi: 10.1186/s40644-026-01072-2 (PMC13520168; doi:10.1186/s40644-026-01072-2)
Supplement: Supplementary file 1 [file 40644_2026_1072_MOESM1_ESM.docx]

| **TABLE S1 Representative information about the acquisition parameters** | | | | | | |
| --- | --- | --- | --- | --- | --- | --- |
| **Sequence** | **TR/TE（msec）** | **FOV**  **(cm²)** | **Matrix** | **Slice thickness (mm)** | **Flip Angle**  **(degree)** | **NEX** |
| 3.0T signa pioneer, GE Healthcare | | | | | | |
| Axial T2WI | 5518/83.2 | 30×34.1 | 288×288 | 5/6 | 110 | 2 |
| Sagittal T2WI | 5938/83.10 | 24×27.3 | 288×288 | 4/5 | 111 | 2 |
| Coronal T2WI | 4811/82.62 | 30×34.1 | 244×320 | 5/6 | 111 | 1 |
| Axial T1WI | 464/7.01 | 32×36.4 | 256×320 | 5/6 | 111 | 1 |
| axial DWI | 4000/75.2 | 34×38.6 | 128×128 | 5/6 | 90 | 6 |
|  | b=0, 1000 s/mm2, ADC map was automatically generated by post process procedure | | | | | |
| DCE | 4.79/1.80 | 36×40.9 | 260×260 | 1.4/1.4 | 15 | 0.7 |
| 3.0T Skyra, Siemens Healthcare | | | | | | |
| Axial T2WI | 3500/85 | 30×34.1 | 288×384 | 4/5 | 120 | 1 |
| Sagittal T2WI | 2520/99 | 24×27.3 | 320×320 | 5/6 | 140 | 1 |
| Coronal T2WI | 5410/85 | 30×34.1 | 288×384 | 5/6 | 120 | 1 |
| Axial T1WI | 547/19 | 24×27.1 | 288×384 | 4/5 | 120 | 1 |
| axial DWI | 5100/51 | 38×43.2 | 95×160 | 4/5 | 90 | 1 |
|  | b=0, 500, 800 s/mm2, ADC map was automatically generated by post process procedure | | | | | |
| DCE | 3.31/1.30 | 38×43.2 | 195×320 | 3/- | 9 | 1 |

T2WI, T2 weighted imaging; T1WI, T1 weighted imaging; DWI, diffusion-weighted imaging; ADC, apparent diffusion coefficient;

DCE, dynamic contrast enhanced
